# Polymorphic Phase Transitions in Carbamazepine and 10,11‐Dihydrocarbamazepine

**DOI:** 10.1002/chem.201802368

**Published:** 2018-08-20

**Authors:** Alexander E. Clout, Asma B. M. Buanz, Simon Gaisford, Gareth R. Williams

**Affiliations:** ^1^ UCL School of Pharmacy University College London 29–39 Brunswick Square London WC1N 1AX UK

**Keywords:** hyphenated techniques, pharmaceutical materials, phase transitions, synchrotron X-ray diffraction, thermal analysis

## Abstract

Temperature‐induced phase transitions in carbamazepine (CBZ) and 10,11‐dihydrocarbamazepine (DHC) were studied by simultaneous differential scanning calorimetry–X‐ray diffraction in this work. The transitions generally involve a transitional melt phase which is quickly followed by recrystallisation. The expansions of the unit cell as a function of temperature could be quantified and allow us to determine a directional order of stability in relation to the lattice constants. Dihydrocarbamazepine form II undergoes a conversion to form I by a localised melt phase. Carbamazepine (CBZ) form IV converts to form I at 182 °C, again by a localised intermediate melt phase. CBZ form II converted to form I at 119 °C by a pathway that appears to have included some melting, and form III underwent a part melt‐recrystallisation and a part sublimation‐recrystallisation to form I.

## Introduction

Pharmaceutical materials may exist in many different physical forms, each of which will have unique physicochemical properties such as solubility, dissolution rate, stability, hygroscopicity, mechanical strength, flowability and compressibility.^1^ All of these properties will have a significant influence on the utility of the compound in a medicine. It is therefore essential that as much as possible is known about the physical form of an active ingredient and how it will behave under different conditions before it can be used in the clinic. However, systemic approaches to understanding polymorphic diversity in organic solids remain elusive.

The standard approach to studying polymorphic transitions is differential scanning calorimetry (DSC). This provides information on the melting point and heat of fusion of polymorphs, and so relative thermodynamic stabilities, but does not give structural information. For the latter, X‐ray diffraction (XRD) is required. Variable‐temperature XRD approaches have led to useful insights into physical form transitions in pharmaceutical materials, but standard lab instruments take a considerable time to record a diffraction pattern (ca. 30 mins) and so it is difficult to study transient or short‐lived phases. Differences in sample sizes between XRD and DSC instruments can also affect the nature of the events observed.^2^ We recently demonstrated that a simple modification to a standard lab DSC instrument permits it to be mounted on a synchrotron X‐ray source, such that diffraction patterns can be obtained in as little as 2 s, as the instrument records thermal data in real‐time.^3^ This allowed us to perform simultaneous DSC–XRD, and collect large amounts of XRD data of a quality suitable for Rietveld refinement during heating at standard DSC rates (10 °C min^−1^). As a result, we could accurately explore and obtain new insights into phase transformations of several pharmaceutical active ingredients, including sulfathiazole, glutaric acid and paracetamol.^3^, ^4^

**Figure 1:**
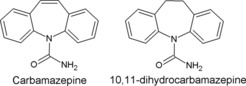
Chemical structures of carbamazepine and 10,11‐dihydrocarbamazepine.

Carbamazepine (CBZ) (Figure 1) is an anticonvulsant commonly used to treat epilepsy and trigeminal neuralgia.^5^ It has extremely poor solubility in water^6^ and is therefore an ideal candidate to screen for metastable polymorphs, since these will have higher solubility and more rapid dissolution rates than the thermodynamically most stable form. To date there have been five CBZ polymorphs reported.^7^, ^8^, ^9^, ^10^, ^11^ CBZ is enantiotropic, and the most stable form at room temperature is form III,^10^ while that at higher temperatures is form I.^9^ The majority of the known polymorphs of CBZ (modifications I–IV) pack as dimers with the two molecules connected *anti* to each other by H‐bonds between the oxygen and nitrogen atoms of the carbamoyl group.^9^, ^11^, ^12^ The differences in structure stem mainly from the way in which these dimers are packed relative to one other. The most recently discovered form (V) is the only known polymorph of CBZ to pack catemerically, without forming dimers. Using DSC, it has been shown that upon heating at 20 °C min^−1^ CBZ II undergoes a solid–solid phase transition to form I between 140 and 160 °C, and form III undergoes a melt‐recrystallisation to form I over the range 168–175 °C.^9^ At the same heating rate form IV melts at 188 °C and partially converts to form I, but slower heating results in a more complete conversion. In all cases form I was shown to melt at 192–194 °C.^9^ The discovery of CBZ V was achieved by templating crystal growth on the surface of a crystal of form II of 10,11‐dihydrocarbamazepine (DHC) (Figure 1). The latter is a structural analogue of CBZ. The difference between the two molecules lies in the presence or absence of a double bond opposite the nitrogen of the azepine ring (Figure 1).

10,11‐dihydrocarbamazepine has four known polymorphs,^13^, ^14^, ^15^, ^16^ three of which display a catemeric H‐bonded motif similar to that of CBZ V. In contrast, the most recently discovered form (IV) has a dimer motif similar to that seen in CBZ forms I–IV. There has been little research into DHC, with no studies reported into the phase transitions between polymorphs or describing their behaviour upon heating.

Here we report, for the first time, detailed studies into the polymorphism of DHC and CBZ, describing a number of new insights into the transformations between the various forms of each.

## Results and Discussion

### Dihydrocarbamazepine

**Figure 2:**
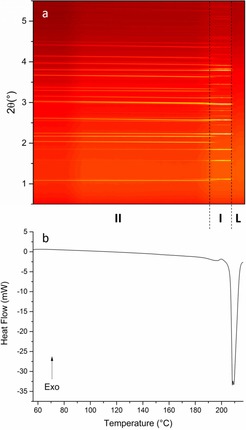
DSC–XRD data for DHC collected at 10 °C min^−1^. (a) A contour plot of the diffraction data showing the phases present: DHC II (II), DHC I (I), and liquid DHC (L). (b) The corresponding DSC thermogram.

Combined DSC–XRD data for a sample of DHC are shown in Figure 2. The XRD data clearly show three distinct regions. In two of these there are numerous Bragg reflections, while the third is conspicuous by its lack of reflections. The DSC thermogram shows no events until the sample reached ca. 190 °C, where there is a small endotherm. At the same temperature there is also a change in the positions of Bragg reflections in the XRD data. Following this, at around 207 °C, there is a much larger endothermic event coinciding with the total loss of Bragg reflections in the contour plot.

Rietveld refinement of a number of DHC crystal structures from the Cambridge Structural Database (CSD) against the pattern recorded for the initial starting material at 56.3 °C (see Supporting Information Figure S1 and Table S1) confirms that it was mostly polymorph II, with a very small amount of polymorph I also present. The refinements fit with a *R*_wp_ of 0.0349, and all subsequent refinements were carried out using the starting models VACTAU01 (form I) and VACTAU02 (form II) from the CSD.

**Figure 3:**
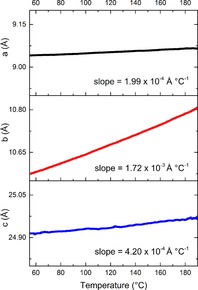
Lattice constants as a function of temperature for 10,11‐dihydrocarbamazepine polymorph II.

**Figure 4:**
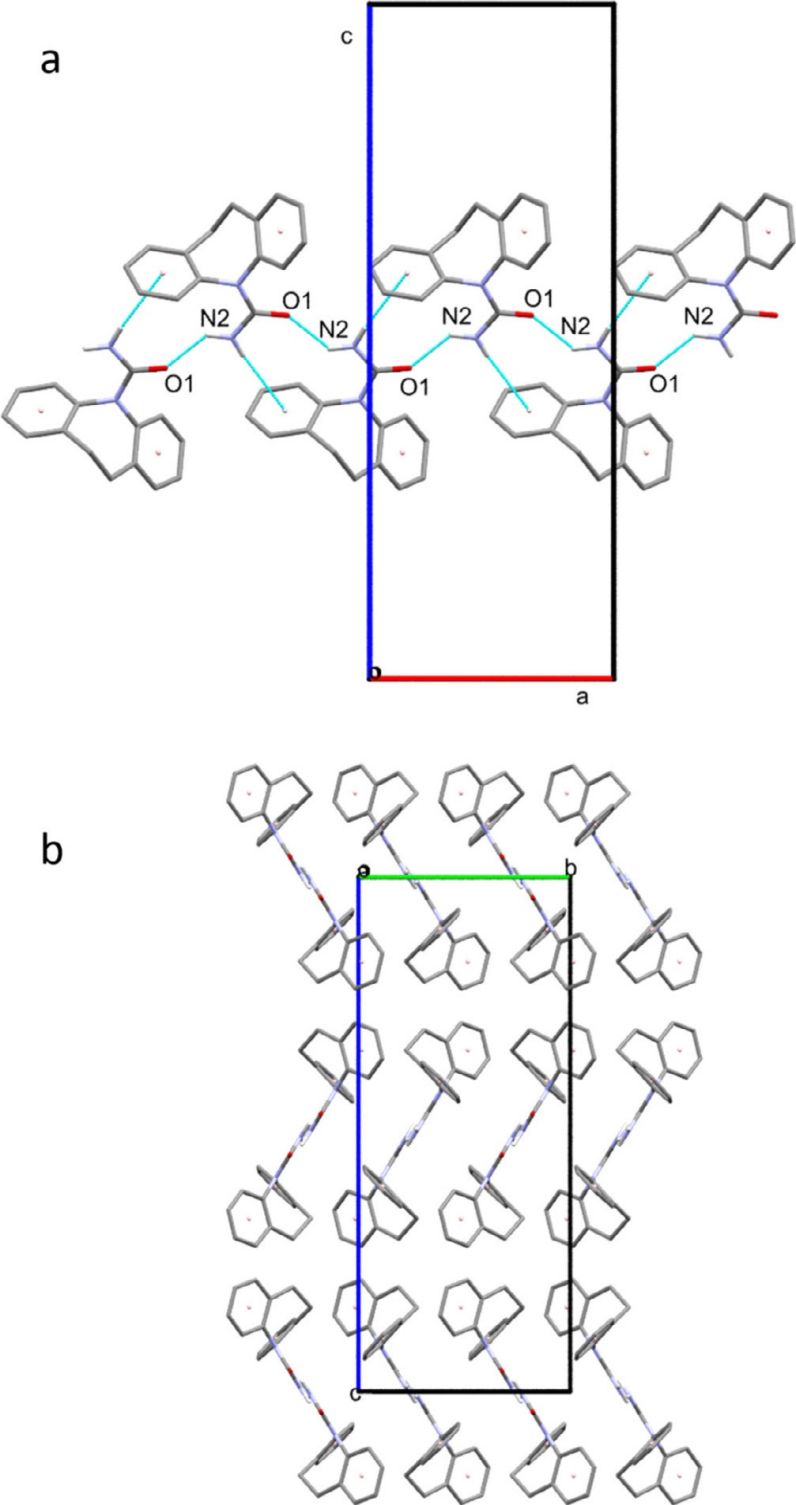
(a) Intermolecular bonding in 10,11‐dihydrocarbamazepine II in the *ac* plane of the unit cell, showing N−H⋅⋅⋅O hydrogen bonds and N−H⋅⋅⋅π interactions. All C‐bound H have been omitted for clarity. (b) 10,11‐dihydrocarbamazepine II viewed in the *bc* plane of the unit cell. All H have been omitted for clarity.

From 56 to 190 °C there are no events in the DSC thermogram, and so there are no phase transitions occurring. However, there are subtle changes in the diffraction data. Some, but not all, of the reflections relating to form II gradually shift to lower 2*θ* angles. This is a consequence of the expansion of the unit cell as it is heated. Figure 3 gives plots of the lattice constants as a function of temperature. The expansion in *b* is larger per degree increase in temperature than that in *a* and *c*, by factors of 9 and 4, respectively. This is a consequence of the alignment of the molecules relative to each other in the crystal and so the distances between them. The dominant intermolecular force in the structure of polymorph II is hydrogen bonding between one hydrogen from the amide group and the oxygen of an adjacent molecule^14^ (Figure S2 and Figure 4 a). This is in fact the only hydrogen bonding throughout the lattice, and while relatively weak for an H‐bond with an H⋅⋅⋅O distance of 2.206 Å^14^, ^17^ is the strongest interaction holding the crystal together. An amide proton of the second molecule forms a similar H‐bond with the oxygen of a third adjacent DHC molecule, and so strings of molecules are formed in one direction. This corresponds exactly to the lattice constant *a*.

The second hydrogen of each NH_2_ group interacts with the benzene ring centroid of the adjacent molecule (Figure 4 a). This enables interaction between the proton and the π‐electrons, which appears to stabilise the one‐dimensional chains.^14^ When viewed in the *ac* plane (Figure 4 a) it is clear that these two interactions stabilise the crystal in the *a* direction.

When viewed in the *bc* plane (Figure 4 b) it can be seen that the chains are arranged in pseudo‐sheets with the orientation of the carbamoyl group alternating with each new layer. Bonding in the *b* direction is much weaker than in any other direction as there are no H‐bonds and only interactions between π electrons of the aromatic rings. Harrison et al.^14^ have suggested that these interactions must be relatively weak as the shortest centroid⋅⋅⋅centroid separation between adjacent molecules is 4.82 Å. Bonding in the *c* axis is a little more interesting. The N−H⋅⋅⋅O H‐bonds between the carbamoyl groups and the supporting N−H⋅⋅⋅π interactions form chains in the *a* dimension and offer some support in *c* (Figure 4 a). Nevertheless, between the chains in this direction there are no hydrogen bonds and only Van der Waals interactions similar to those in the *b* axis, but slightly stronger, with the shortest C⋅⋅⋅C separation being 3.651 Å. This intermolecular bonding structure fits well with the unit cell expansion pattern observed, with the greatest expansion seen in *b*, followed by *c* and finally *a*.

**Figure 5:**
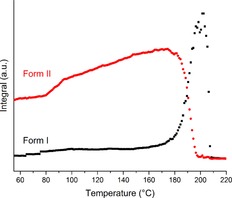
Plot of integrated total diffracted intensity for calculated patterns of 10,11‐dihydrocarbamazepine form I (black squares) and form II (red circles) as a function of temperature.

At 190 °C the form II reflections fade away and new ones grow in at different angles; a small endotherm also appears in the thermogram. These two events indicate a phase transition. Rietveld refinement of the diffraction pattern recorded at 202.5 °C (Table S1 and Figure S3) demonstrates that the second phase is DHC I. Thus, the phase transition occurring is form II converting to form I. It is notable that the presence of crystalline material in the beam was constant. The results of integration of the calculated patterns for the two forms as a function of temperature are presented in Figure 5. It is clear that the initial sample consisted almost entirely of form II. As the temperature rises the content of form II appears to increase, whilst the amount of form I remains relatively constant. One explanation for the apparent growth of form II may be that there was some amorphous material present in the initial sample and that the energy supplied upon heating allowed sufficient molecular mobility for crystallisation. However, glassy DHC would produce a broad shallow “hump” rather than sharp peaks in a diffraction pattern. Further inspection of the initial pattern (Figure S1) reveals no indication of the presence of amorphous material. There is a visible curve to the background but this persists throughout all of the data collected on all of the samples during this beamtime. More significantly, there are no exothermic events in the DSC trace, ruling out any rapid crystallisation occurring. It could be that the increase in form II arises because of gradual crystallisation occurring during the heat (below the detection limit of the DSC) or it may be a consequence of the expansion of crystals in the sample resulting in more material being lifted from the bulk into the passing beam.

At around 150 °C the amount of form I begins to increase. Following this, at 175 °C, the amount of form II begins to decrease and the rate of growth of form I rises sharply. The growth and decay of these two crystal structures continue in an approximately linear fashion until the sample reaches 200 °C and there is no more form I present. The small endotherm in the calorimetric data covers the same temperature range and the combination of these results indicates the occurrence of a phase transition from form II to form I. The crossing of the two curves at around half the maximum quantity of either of the two species indicates the transition does not occur via a complete melt of the sample. Instead it is likely a solid–solid transition, or possibly the result of many smaller melt‐recrystallisation events on a particle by particle basis. The absence of an exotherm in the thermogram and the presence of the endotherm suggests the latter is perhaps more likely (although it could be that exothermic and endothermic events are happening concurrently, resulting in a net endotherm). It is likely that the presence of some form I in the initial sample seeded the process. Analysis of the gradient of the two curves has shown the decline of form II content to be −0.78 °C^−1^ and the growth of I to be 0.81 °C^−1^ between 191 and 196 °C. The similarity between these two numbers indicates that the conversion was a single‐step process.

Following the II→I conversion, crystalline material is only present over a temperature range of around 10 °C before a total loss of reflections is observed in the XRD data. The large endotherm in the DSC trace at 207 °C confirms that the sample has melted.^18^ Although polymorph I was not present for long, it was nevertheless possible to extract the lattice constants and cell volume from the refinements and plot them as a function of temperature (Figure S4).

As with form II the unit cell expands in three dimensions as the temperature increases. Expansion in *c* per degree temperature rise is greater than that in *a* and *b* by factors of 3.5 and 7 respectively. The reasons for this are similar to those for form II. Form I exhibits the same molecular chains held together by weak hydrogen bonding between an amide proton and the oxygen of an adjacent molecule^13^, ^14^ and stabilised by N−H⋅⋅⋅π interactions between the second amide proton and an adjacent benzene centroid, but instead of the *a* direction they propagate along *b* (Figure S6). When viewed in the *ac* plane (Figure S7) the difference between the two structures is clear. In both, the chains are arranged in pseudo sheets, but where in form II the orientation of the carbamoyl groupings alternates with each layer (Figure 4 b), form I presents them in the same orientation. In both forms the layers are positioned so that the ring structures of each molecule are adjacent to ring structures in another molecule, allowing for π⋅⋅⋅π interactions.

For both DHC I and II, the smallest expansion is observed in the same direction as the propagation of the chains. This is to be expected as this is the direction in which the strongest intermolecular interactions are observed. However, the greatest expansion and so the weakest interactions are seen in different relative directions. Form II expands most in *b* (equivalent to *a* in form I), effectively increasing the area of the pseudo sheets, whereas form I expands most in *c* (equivalent to *c* in form II), increasing the space between the pseudo sheets. Both of these dimensions are dominated by π⋅⋅⋅π interactions, which are weaker than H‐bonds.^19^, ^20^

### CBZ IV

**Figure 6:**
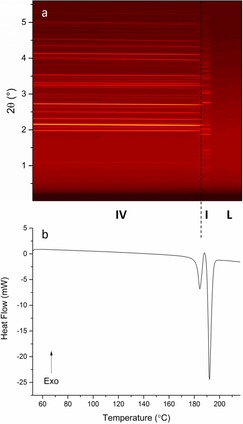
DSC–XRD data for CBZ IV collected at 10 °C min^−1^. (a) A contour plot of the diffraction data and (b) the corresponding DSC thermogram.

There has been some confusion over the nomenclature of CBZ polymorphs in the literature; in this work, we use the numbering of the CSD (Table 1). DSC–XRD data for a sample of anhydrous CBZ supplied as form IV can be seen in Figure 6. The diffraction data are similar to those of DHC, with the occurrence of one crystalline to crystalline and one crystalline to liquid transition. Data collection began at 52 °C and no major structural changes occurred until 182 °C, at which point there is a change in the 2*θ* positions of the Bragg reflections. The second crystalline phase is present until the sample reaches around 192 °C, at which point all reflections disappear. The DSC trace shows a small endotherm–exotherm event coinciding with the crystalline–crystalline transition. Immediately following this and superimposed upon it there is a much larger endotherm, resulting from melting of the material (evident from the total loss of diffracted intensity at the same temperature). The proximity of these events indicates that, at a heating rate of 10 °C min^−1^, the two transitions occur at very similar temperatures. Unfortunately, a consequence of the overlapping thermal events is that there can be no accurate quantification of the associated enthalpies.

**Table 1:** Unit cell information and CSD identifiers for CBZ forms I–IV.

Property	Form I	Form II	Form III	Form IV
CSD identifier	CBMZPN11^9^	CBMZPN03^8^	CBMZPN01^7^	CBMZPN12^10^
*T* [°C]	−115	10–30	10–30	−115
space group	*P* $\bar{1}$	*R* $\bar{3}$	*P*2_1_/*c*	*C*2/*c*
*a* [Å]	5.1705(6)	35.454(3)	7.529(1)	26.609(4)
*b* [Å]	20.574(2)	35.454(3)	11.148(2)	6.9269(10)
*c* [Å]	22.245(2)	5.253(1)	15.470(2)	13.957(2)
*α* [°]	84.124(4)	90	90	90
*β* [°]	88.008(4)	90	116.17(1)	109.702(2)
*γ* [°]	85.187(4)	120	90	90
*R* factor	0.0506	0.069	0.035	0.0357

Batch Rietveld refinement was carried out on all patterns recorded and selected patterns were examined individually. Initially, selected patterns were analysed to establish which of the five reported forms of CBZ^8^, ^9^, ^10^, ^11^, ^21^ were present throughout the experiment. These analyses found evidence of forms I and IV but no other species. All further refinements were carried out using starting models from the CSD (form I: CBMZPN13, form IV: CBMZPN12). Unit cell data are presented in Table S2. Refinement of the initial pattern recorded at 52 °C and a pattern recorded at 189 °C are given in Figure S8. Evidently, the initial sample was entirely form IV, and the structural refinements fit with a *R*_wp_ of 0.0441.

Although CBZ is analogous to DHC with some of its polymorphs having similar structures to those of DHC already discussed, the structure of form IV (Table 1) is not one of these. Unsurprisingly, plotting the lattice parameters as a function of temperature (Figures S9 and S10) reveals that the unit cell of form IV expands upon heating, with expansion in *b* being around twice that of *a* and *c*. CBZ IV packs as dimers, held by two H‐bonds (1.86 Å) through the carboxamide group with the two molecules anti to each other^10^ (Figure S11). The oxygen also takes part in another interaction (2.28 Å) with a hydrogen on the seven‐membered ring of an adjacent molecule, and so chains of molecules are formed, which propagate along *c* (Figure S12). These chains are held together along *a* and *b* by centroid‐centroid interactions at a distance of 3.809 Å. The bonding pattern in *a* alternates between the two H‐bonds forming the dimers and centroid‐centroid interactions linking each dimer with the next (Figure S13).

**Figure 7:**
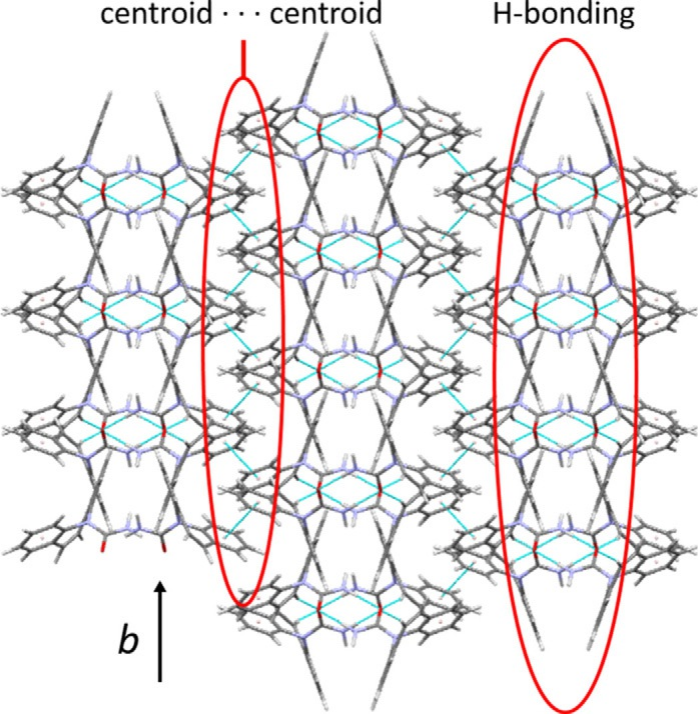
Intermolecular bonding in CBZ IV along the *b* axis of the unit cell, with *c* perpendicular to the page, showing N−H⋅⋅⋅O H‐bonds within dimers and C−H⋅⋅⋅O H‐bonds and centroid⋅⋅⋅centroid interactions between dimers.

Figure 7 shows the three types of intermolecular bonds present throughout the crystal. It is immediately apparent that in the *b* axis the H‐bonds exist as individual units and offer very little support in this dimension. As a result, bonding in this direction is dominated by benzene ring interactions; while these do form very clear chains, the interactions in them are much weaker than in the other two dimensions. This becomes clear when comparing the distances between the interacting species (N−H⋅⋅⋅O, 1.86 Å; C−H⋅⋅⋅O, 2.28 Å; centroid⋅⋅⋅centroid, 3.809 Å; π⋅⋅⋅π offset, 1.206 Å).

Clearly the domination of H‐bonding in *c* makes the interactions in this direction stronger than *a*, which has a combination of both H‐bonds and π⋅⋅⋅π interactions. Axis *a* in turn has stronger intermolecular interactions than *b*, which exhibits almost entirely π⋅⋅⋅π interactions. As a result, expansion in the *a* axis is slightly larger (1.39×) per °C than that in the *c* axis and that in *b* is larger than *a* and *c* by factors of 2.35 and 3.26 respectively.

**Figure 8:**
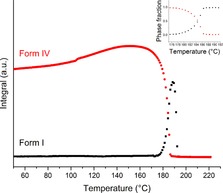
Plot of integrated total diffracted intensity for calculated patterns of CBZ I (black squares) and IV (red circles) as a function of temperature, with inset plot of phase fraction as a function of temperature.

Subsequent to this expansion of the unit cell the data show a small endotherm‐exotherm and a sharp change in profile of the diffraction patterns. The initial small endotherm has an onset of 180 °C and represents the melting of form IV. The exotherm denotes a recrystallisation process. Rietveld refinement of the pattern recorded at 189 °C after the profile change and the peak of the exotherm (Figure S8) identifies the second phase as CBZ polymorph I, with a residual trace of IV remaining. Plotting the integrated total diffracted intensity for each pattern as a function of temperature (Figure 8) reveals that the lack of form I below 170 °C is constant and the amount of form IV seems to grow as the temperature increases. This is likely due to thermal effects. The total integrated intensity peaks at 152 °C, at which point it begins to decrease before a very sharp drop which flattens out at 192 °C. This signifies the total loss of all form IV content in the sample. At 172 °C form I begins to grow; this is around the same point at which the decrease in form IV accelerates. The two changes considered together suggest that form IV converts to form I upon heating.

The integrated data were converted to phase fractions and plotted as a function of time (Figure 8 inset). The curves cross at 0.5 and so the transition occurred without any wholesale melt (i.e. melting and recrystallisation occur concomitantly rather than sequentially). An approximate linear fit of the integrated data has been carried out around the intersection point. It appears that the decline of form IV (−5.100 °C^−1^) occurs at a slightly faster rate than the evolution of form I (3.567 °C^−1^). This is unsurprising as melting is a thermodynamic event and occurs very quickly while crystallisation is a kinetic event requiring molecular ordering. The presence of the endotherm‐exotherm in the DSC trace (Figure 6) offers strong support to the theory of phase transformation via melting. The reason for their overlap is that the temperature at which the material crystallises to I is reached by the instrument before all of form IV has melted. It appears that the transformation of CBZ IV to CBZ I must occur by a melt‐recrystallisation mechanism at these heating rates. The possibility of a separate melt and recrystallisation occurring at a lower heating rate cannot be ruled out.

Following the conversion of CBZ IV to I it can be seen that the maximum form I content barely reaches 70 % of the maximum form IV content prior to the conversion (Figure 8), as a result of incomplete recrystallisation to form I. At 10 °C min^−1^ the DSC reached the melting temperature of form I^22^ (represented by the large endotherm, onset 191 °C in Figure 6 b) before the material had all been able to crystallise. When the experiment was repeated at 2 °C min^−1^ (data not shown) the endotherm and exotherm were no better resolved but the resultant crystallisation exotherm had time to complete prior to the onset of melting and the total content of form I after the conversion had completed was similar to that of form IV. This agrees with work by Grzesiak et al.^9^ Presumably at the faster heating rate, the melted form IV remains in the molten state until the end of the experiment.

### CBZ II

**Figure 9:**
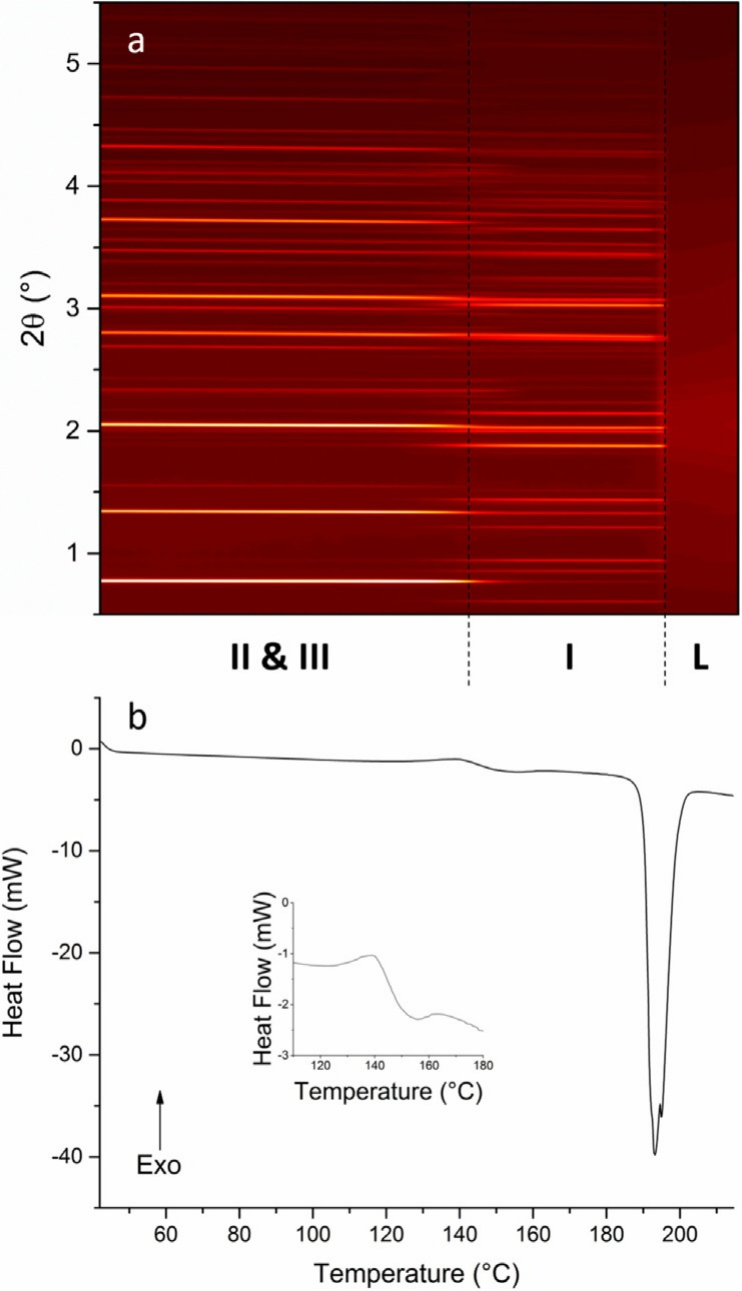
DSC–XRD data for a sample of carbamazepine procured as form II, collected at 10 °C min^−1^. (a) A contour plot of the diffraction data and (b) the corresponding DSC thermogram. The inset in (b) shows a magnified view of DSC events between 110 °C and 180 °C. Dashed lines indicate the position of phase transitions visible to both DSC and XRD.

DSC–XRD data for a sample of carbamazepine supplied as form II are given in Figure 9. The DSC data show a small exotherm–endotherm with an onset at 126 °C, and then a large endotherm with two peaks and an onset at 190 °C. The former corresponds to a complete change in the diffraction pattern, while the latter is concurrent with the complete loss of all Bragg reflections and is in agreement with the reported melting point of CBZ form I.^22^, ^23^ The reason for the double peak is unclear, but it may be explained by the large sample size (12.1 mg). In all DSC–XRD experiments it was necessary to use a sample size significantly larger than the 5 mg recommended, to ensure that there was always sample in the X‐ray beam.

The initial change in diffraction pattern is accompanied by an exotherm‐endotherm in the DSC trace, indicating crystallisation followed by melting. Following the transition some crystalline material remains, but a portion appears to have melted. This suggests that the initial sample may have been a mixture of polymorphs rather than pure form II. Furthermore, form II is reported to undergo exothermic conversion to form I between 140 and 160 °C at a heating rate of 20 °C min^−1^.^9^ The onset of the exotherm in this experiment occurs at 119 °C, which can be accounted for by the slower heating rate (10 °C min^−1^), but the endotherm cannot be attributed to the same conversion. Unfortunately, the onset occurs whilst the preceding exotherm is ongoing and so cannot be accurately determined, but it must be above 119 °C. Additionally, due to the small enthalpy of the endotherm it is unclear whether the signal subsequently returns to baseline or if there is another exotherm prior to the melt.

Phase identification was carried out on patterns collected at 42 and 178 °C using the Rietveld method. A relatively poor fit was obtained at 42 °C when considering only form II in the model, leading to a more detailed analysis in which the structures of all five reported polymorphs were introduced. The conclusion of these refinements was that the initial sample was in fact a mixture of forms I (3.5 %), II (87.8 %) and III (8.7 %) (Figure S14a). All further refinements were carried out using starting models from the CSD (summarised in Table 1). Upon closer inspection of the pattern recorded at 42 °C (Figure S14a) it appeared that all form I content was attributable to a shift in the background and that there were no specific reflections assigned to that structure; as a result, we determined that the detection of form I was an artefact and it was excluded from refinements at low temperature. Refinement of the higher temperature pattern (Figure S14b) revealed that following the phase transition almost all of the material had converted to form I (96 %) with a very small quantity of form II (2 %) and III (2 %) remaining. However, as with form I in the low temperature pattern, there were no characteristic reflections of form III remaining in the observed data at 178 °C and the 2 % can again be accounted for by a slight discrepancy in the background. Final unit cell data for the patterns discussed are presented in Table S3.

Batch refinements were carried out and fits were obtained with *R*_wp_ values between 0.0944 and 0.1401. Figures S15–S18 show plots of lattice constants as a function of temperature for the three forms of CBZ present. As with all previous materials, increasing the temperature of the sample causes expansion of the unit cell in all three dimensions for all three polymorphs. In all three structures the molecules pack as very similar dimers to those already discussed for CBZ IV (Figure 7 and Figure S11), held by intermolecular H‐bonds between the carboxamide groups. Polymorph II expands over 5 times more per °C in the *a* and *b* dimensions than it does in the *c* dimension. This is because the dimers in the structure arrange to form pseudo‐layers in the *ab* plane.^8^ These layers stack with translational symmetry that runs parallel to the *c* axis. It is clear from these data that the sum of the intermolecular bonding in the *c* axis is much stronger than in the other dimensions, resulting in the broadening of the layers as the sample is heated, whilst the chains of stacked dimers increase in length more slowly.

Expansion differences between the three lattice constants of form III are far less pronounced than those of form II. The intermolecular bonding in this structure consists of the H‐bonds forming dimers, two centroid⋅⋅⋅centroid interactions of slightly different lengths, two C−H⋅⋅⋅O interactions, a N−H⋅⋅⋅π contact and a C−H⋅⋅⋅π interaction.^24^ This combination of intermolecular bonding is complex and has clearly led to less H‐bond dominance throughout the structure, hence the more uniform expansion of the unit cell upon heating.

**Figure 10:**
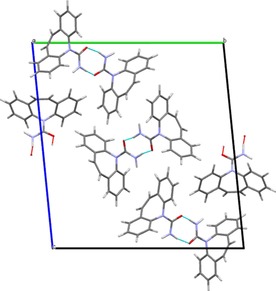
Graphic representation of the unit cell of CBZ I, showing H‐bonds.

Intermolecular bonding in polymorph I is also complex and has many similarities with form III. In both forms the oxygen is involved in dimer formation as well as interactions with the vinylic carbons of adjacent molecules. A more detailed description is given by Grzesiak and co‐workers.^9^ Using this description it was possible to examine the packing of the crystal using Mercury 3.8. When only the H‐bonds which form the dimers are considered (Figure 10), it can be seen that three of the four pairs of bonds are orientated so that they offer support along *b* and only one of the pairs offers support to *c*. As these are the dominant intermolecular interactions in the crystal this explains the much larger extent of expansion in *c*.

As can be seen from the contour plot (Figure 9), the presence of solid crystalline material in the path of the beam was constant from 42 to 196 °C. However, as the initial sample was a mixture of two polymorphs it cannot be determined from this plot alone whether either of the components melted at any point.

**Figure 11:**
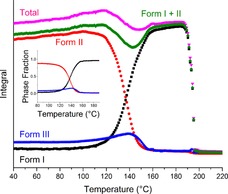
Plot of integrated total diffracted intensity for the calculated patterns of CBZ I (black squares), II (red circles), III (blue triangles), their sum (pink triangles) and CBZ I+II (green diamonds) as a function of temperature, with an inset plot of phase fraction as a function of temperature.

Figure 11 shows the amount of each polymorph present in the sample as a function of temperature. Below 100 °C the relative contributions of the three polymorphs is constant, with no form I, a little form III and the sample consisting mainly of form II. As with DHC and CBZ IV the total content of form II appears to increase with the temperature until it reaches 100 °C, at which point the first phase transition begins. This increase is probably a result of thermal expansion. Form I begins to grow in at around 100 °C and the gradients of both curves become much steeper at 120 °C. This coincides with the onset of the exotherm in the DSC trace (119 °C) and suggests crystallisation from II to I. A linear fit was carried out at the straightest section of the curves around the point at which they cross (132–144 °C) and the rate of decay of form II (−3.794 °C^−1^) and the rate of growth of form I (3.164 °C^−1^) are similar, with form I growing a little more slowly, as is to be expected when comparing crystallisation (kinetic) to melting (thermodynamic).

During this time form III appears to grow at an increasing rate until its content peaks at around 140 °C and immediately begins to decay. It is difficult to say whether form II melts or converts directly to form I; however, the presence of the endotherm in the DSC data suggests a melt. The crossing of the phase fraction curves at approximately 0.5 means that this is not a wholesale melt, but instead localised melting could be occurring. A plot of the sum of the calculated contributions of forms I and II shows a dip in the crystalline content in the beam between 120 and 160 °C, during the phase transition and prior to the waning of form III. This supports the hypothesis that there is some melting of form II involved in the transformation, but disagrees with the literature, which describes a solid–solid transition (based on a combination of thermomicroscopy and DSC carried out on separate samples).^9^ Presumably there must be a point during the transition at which the structure of the material sits somewhere between those of the two crystals and is disordered, unless the conversion is simultaneous and instantaneous for all molecules in the crystal lattice; this may be what causes the drop in overall crystalline content at ca. 140 °C, and may not strictly be described as a melt.

Plotting the sum of the content of the three polymorphs as a function of temperature (Figure 11) it can be seen that the total crystalline content is lower following the phase transition. A thorough investigation of the thermal relationship between forms I and III was carried out by Behme and Brooke^25^ who concluded that the heating rate has a strong effect on the behaviour of form III. At 2 °C min^−1^ there is sufficient time allowed for the full conversion to form I, via a sublimation‐condensation mechanism between 150 and 170 °C, with no apparent melting. However, once increased to 10 °C min^−1^ they state that “the endotherm recorded in the range 165–175 °C reflects several thermal events”, representing the combination of some conversion to form I via sublimation and some melting. At all rates studied in the range 2–40 °C min^−1^ the endotherm was followed by an exotherm corresponding to the crystallisation of form I from the melt. This melt‐recrystallisation pathway is well documented in the literature.^9^, ^22^, ^23^, ^25^, ^26^, ^27^ It is likely that the drop in overall diffracted intensity is a result of some form III subliming and moving out of the incident beam whilst in the gas phase and condensing in another area of the sample or being lost from the pan, which was left open for these experiments. Hence, it seems that there are two distinct phase transitions occurring simultaneously by three mechanisms. Form II is undergoing melt–recrystallisation to form I on a microscopic particle by particle basis and form III is undergoing part sublimation‐recrystallisation and part melt‐recrystallisation to form, I.

The conversion data presented here are consistent with the literature, except that the conversion of III occurs at a lower temperature than expected. This may be due to the presence of form II destabilising it. Due to the overlapping events in the thermogram and the open pan it is impossible to quantify the enthalpy of conversion for either the form III→I or II→I transitions. Consequently, the amount of form III lost by sublimation cannot be determined via enthalpic calculations. However, the diffraction data were used to calculate a rough fraction of III lost by calculating the difference between the maximum total crystal content prior to the phase transition and the equivalent immediately following it. This was compared to the maximum area under the curve for form III and the resulting Figure suggests that only 30 % recrystallised to form I following the melt and 70 % was lost from the beam. This may be partially prevented in future work by using hermetically sealed DSC pans; however, this would not prevent recrystallisation from the vapour phase in areas of the sample not interrogated by the incident beam.

## Conclusions

Temperature‐induced phase transitions in carbamazepine (CBZ) and 10,11‐dihydrocarbamazepine (DHC) were studied by simultaneous DSC–XRD in this work. The transitions generally involve a transitional melt phase. This melt is so quickly followed by recrystallisation that regions of the sample melt and recrystallise before other regions begin to melt. Consequently, the presence of crystalline material in the sample is continuous, leading to the assumption of solid–solid transitions in the previous literature. Batch Rietveld refinements allowed the expansion of the unit cell to be quantified as a function of temperature, and these data in conjunction with structural information allow us to determine a directional order of stability in relation to the lattice constants. The dimensions which contain stronger intermolecular bonding (e.g. H‐bonds) have shown smaller expansion per °C than those with weaker interactions (e.g. π⋅⋅⋅π). DHC II was shown to undergo a conversion to form I by what appears to be a localised melt phase. CBZ IV converted to form I at 182 °C, again by a localised intermediate melt phase. A sample procured as pure CBZ II was in fact found to contain a small amount of form III. Form II converted to form I at 119 °C by a pathway that appears to have included some melting, and form III underwent what seems to be a part melt–recrystallisation and a part sublimation‐recrystallisation to form, I.

## Experimental Section

### Materials

Carbamazepine (98 %) forms II and IV were provided by Dr. Vijay Srirambhatla (EPSRC CMAC Future Manufacturing Research Hub, University of Strathclyde); 10,11‐dihydrocarbamazepine (99 %) was purchased from Alfa Aesar, UK and used as received.

### DSC–XRD

DSC Measurements were performed with modified TA 2010 or Q20 instruments (TA Instruments LLC), with holes drilled in the furnace to permit the passage of the X‐ray beam as detailed in our previous study.^3^ Calibration was performed with a certified indium standard according to the manufacturer's instructions. Samples of all materials (5–20 mg) were held in Tzero aluminium pans and heated at 10 °C min^−1^ from ambient to 220 °C. Experiments were performed on Beamline I12 of the Diamond Light Source using a 0.5×0.5 mm beam of monochromated X‐rays at 52.4 keV (0.236 Å). A Thales Pixium RF4343 detector, calibrated with a CeO_2_ standard, was located 2.4 m away from the sample. Diffraction patterns were recorded every six seconds (data were collected for 4 s with a 2 s pause between collections).

### Data analysis

The DAWN Science Workbench was first used to convert the 2D data into 1D diffraction patterns.^28^ Contour plots of the raw XRD data were then plotted using OriginPro 2016. Selected patterns were analysed using the Rietveld method implemented within the TOPAS‐Academic suite of programmes,^29^ in order to obtain realistic values for the unit cell parameters at elevated temperatures. Backgrounds were fitted using a shifted Chebyshev polynomial of the first kind with between 6 and 15 terms. Lattice parameters and peak shape parameters were refined. In cases where more than one phase was present, the peak shapes for each phase were constrained to be the same and the phase fraction was refined. The models used came from the CCDC (details are given above). The atom positions were not refined. Atom displacement parameters, *U*_iso_ were set to be 0.15 Å^2^ in each phase. Once starting parameters were obtained batch refinements were performed on all datasets collected. No zero point was refined as entire diffraction patterns were collected using a 2D area detector.

## Conflict of interest

The authors declare no conflict of interest.

## Supporting information

As a service to our authors and readers, this journal provides supporting information supplied by the authors. Such materials are peer reviewed and may be re‐organized for online delivery, but are not copy‐edited or typeset. Technical support issues arising from supporting information (other than missing files) should be addressed to the authors.

Supplementary
