# Supplementary material for: Polymorphic Phase Transitions in Carbamazepine and 10,11‐Dihydrocarbamazepine
Source: Chemistry. 2018 Aug 20;24(51):13573–81. doi: 10.1002/chem.201802368 (PMC6175174; doi:10.1002/chem.201802368)
Supplement: Supplementary file 1 — Supplementary [file CHEM-24-13573-s001.pdf]

CHEMISTRY
A European Journal

Supporting Information

Polymorphic Phase Transitions in Carbamazepine and 10,11-Dihydrocarbamazepine

Alexander E. Clout, Asma B. M. Buanz, Simon Gaisford,* and Gareth R. Williams*[a]

chem_201802368_sm_miscellaneous_information.pdf

Supporting information

Table S1. Refinement parameters for 10,11-dihydrocarbamazepine at 56.3 °C and 202.5 °C. The starting models were taken from the CSD (form I: VACTAU01; form II: VACTAU02).

Property	Form II	Form I
T / °C	56.3	202.5
Space group	*Pbca*	$P2_1/c$
a / Å	9.0412(9)	5.5573(2)
b / Å	10.5748(8)	9.1969(6)
c / Å	24.914(2)	24.433(1)
α / °	90	90
β / °	–	96.505(4)
γ / °	90	90
Cell volume / Å^3	2382.0(5)	1240.7(1)
R_{wp}	0.0349	0.0440
$R_{wp\text{-}bkd}$ **	0.1487	0.1944
Phase fraction*	94.7 %	99.0 %

* Due to the graininess of the sample, representative errors cannot be calculated. However, the authors conservatively estimate the error to be in the region of 5 %. ** $R_{wp\text{-}bkd}$ is the R_{wp} calculated excluding the background fit.

Table S2. Refinement parameters for carbamazepine IV. The starting models were taken from the CSD (form IV: CBMZPN12; form I: CBMZPN11).

Property	Form IV	Form I
T / °C	52.2	188.5
Space group	*C2/c*	*P21/c*
a / Å	26.517(1)	5.3585(3)
b / Å	7.076(0)	20.522(1)
c / Å	13.949(1)	22.472(2)
α / °	90	84.318(6)
β / °	109.578(5)	87.363(6)
γ / °	90	85.540(5)
Cell volume / Å^3	2465.9(2)	2449.8(3)
R_{wp}	0.0441	0.0401
$R_{wp\text{-}bkd}$	0.1671	0.2071
Phase fraction*	98.8 %	98.4 %

* Due to the graininess of the sample, representative errors cannot be calculated. However, the authors conservatively estimate the error to be in the region of 5 %. ** $R_{wp\text{-}bkd}$ is the R_{wp} calculated excluding the background fit.

Table S3. Refinement parameters for carbamazepine II. The starting models were taken from the CSD (form I: CBMZPN13; form II: CBMZPN03; form III: CBMZPN26).

Property	Form II	Form III	Form I	Form II
T / °C	42		178	
Space group	*R*-3	$P2_1/n$	*P*-1	*R*-3
a / Å	34.830(1)	7.515(3)	5.3435(8)	35.25(4)
b / Å	-	11.128(4)	20.510(3)	-
c / Å	5.3209(4)	13.883(9)	22.440(3)	5.372(12)
α / °	90	90	84.272(13)	90
β / °	90	92.85(4)	87.440(18)	90
γ / °	120	90	85.476(16)	120
Cell volume / Å^3				
R_{wp}	0.1301		0.1337	
$R_{wp\text{-}bkd}$	0.2324		0.2438	
Phase fraction	91.0 %	9.0 %	98.0 %	2.0 %

* Due to the graininess of the sample, representative errors cannot be calculated. However, the authors conservatively estimate the error to be in the region of 5 %. ** $R_{wp\text{-}bkd}$ is the R_{wp} calculated excluding the background fit.

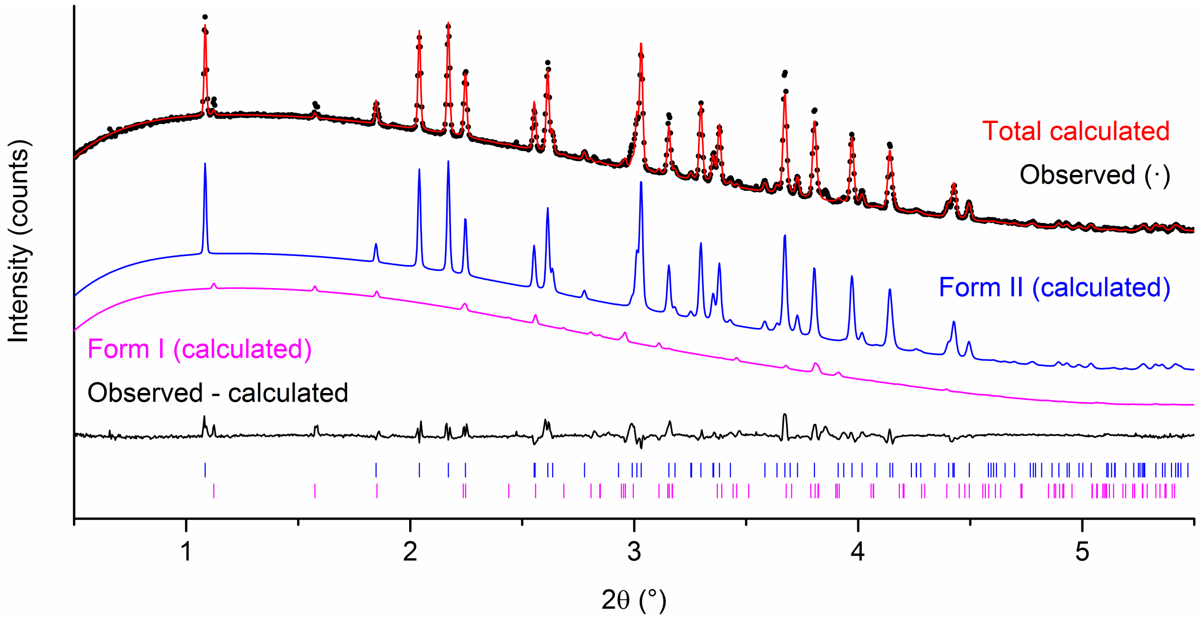

Figure S1. Rietveld refinement data for 10,11-dihydrocarbamazepine at 56 °C. Tick marks show the positions of allowed reflections of 10,11-dihydrocarbamazepine form II (upper) and form I (lower).

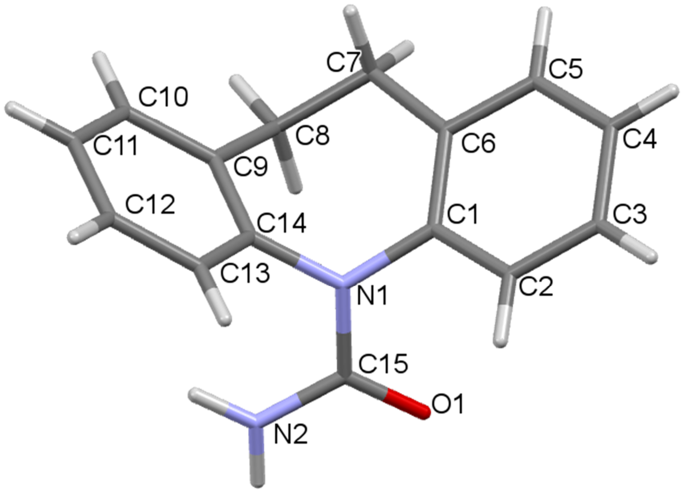

Figure S2. Molecular structure of 10,11-dihydrocarbamazepine with atoms numbered for ease of identification. [Image produced using Mercury 3.8].

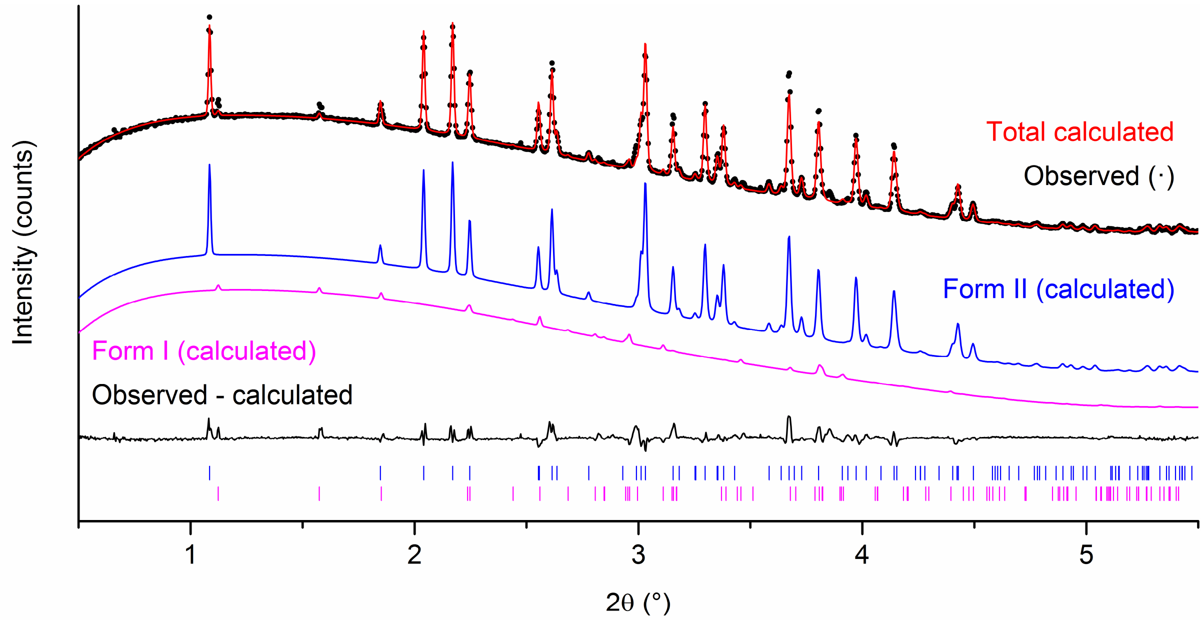

Figure S3. Rietveld refinement data for a diffraction pattern recorded for DHC at 202.5 °C. The structure fits the data with a R_{wp} of 0.0440. Tick marks show the positions of allowed reflections of DHC form II (upper) and form I (lower).

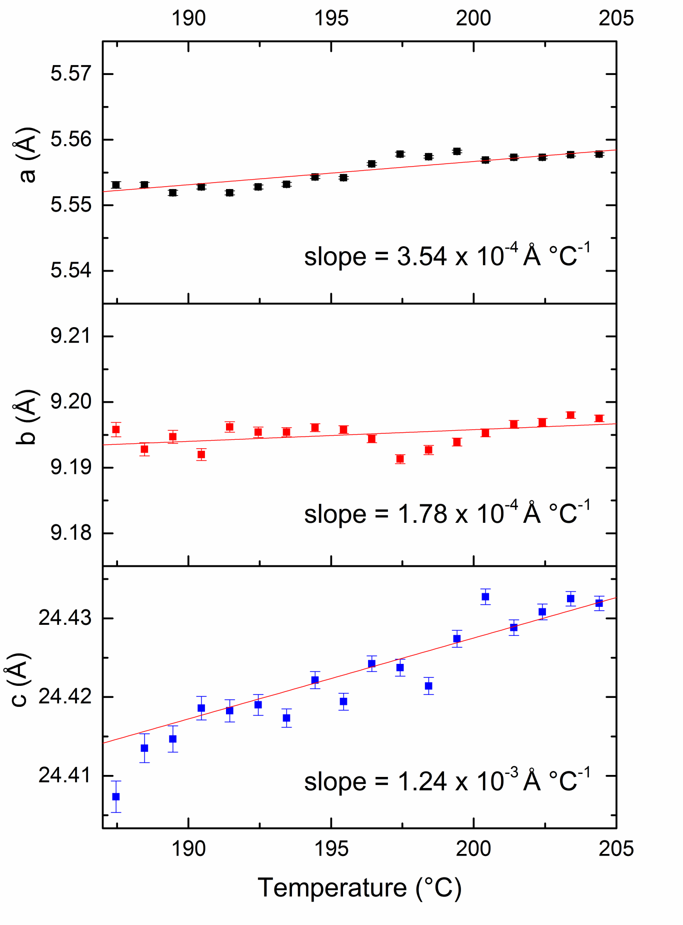

Figure S4. Lattice constants as a function of temperature for 10,11-dihydrocarbamazepine polymorph I.

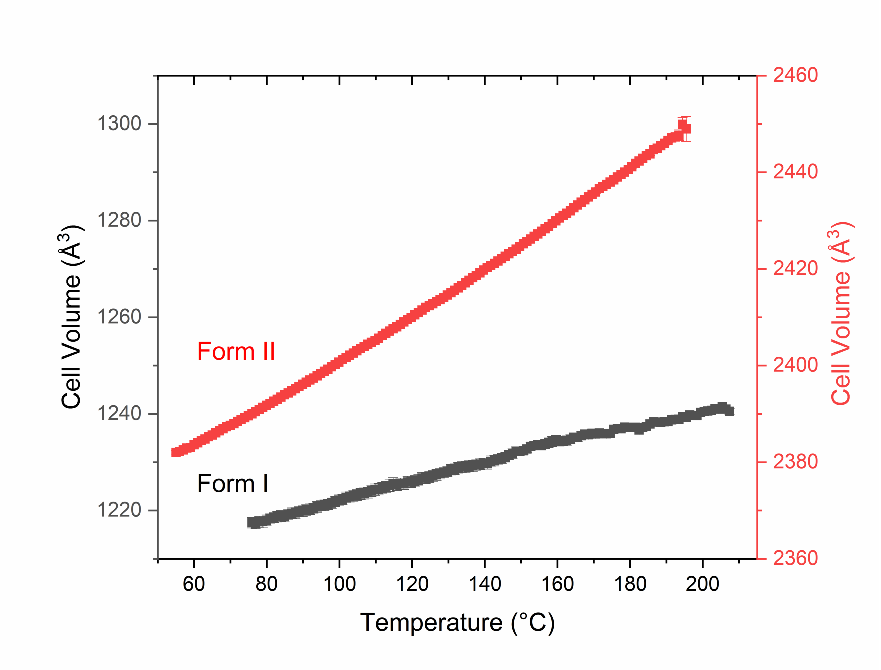

Figure S5. Cell volume as a function of temperature for 10,11-dihydrocarbamazepine polymorphs I (black) and II (red).

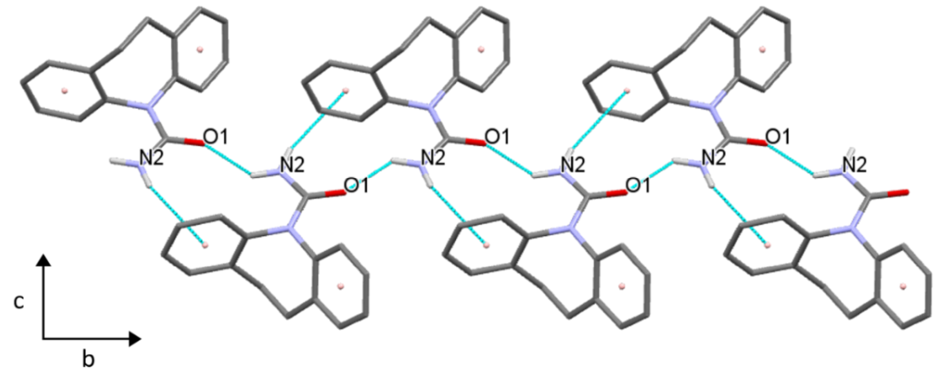

Figure S6. Intermolecular bonding in 10,11-dihydrocarbamazepine I in the *bc* plane of the unit cell. Showing N—H···O hydrogen bonds and N—H···π interactions. All C bound H have been omitted for clarity.

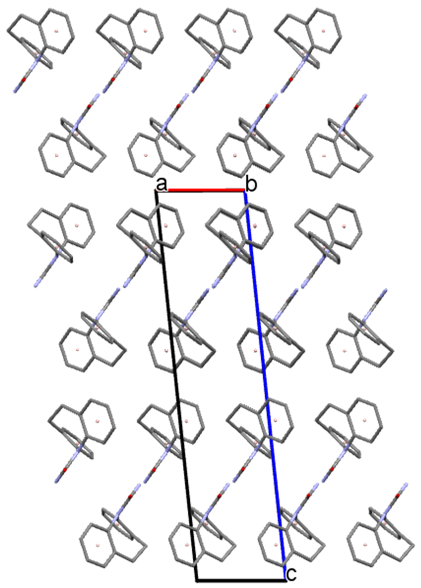

Figure S7. 10,11-dihydrocarbamazepine I viewed in the *ac* plane of the unit cell. All H have been omitted for clarity.

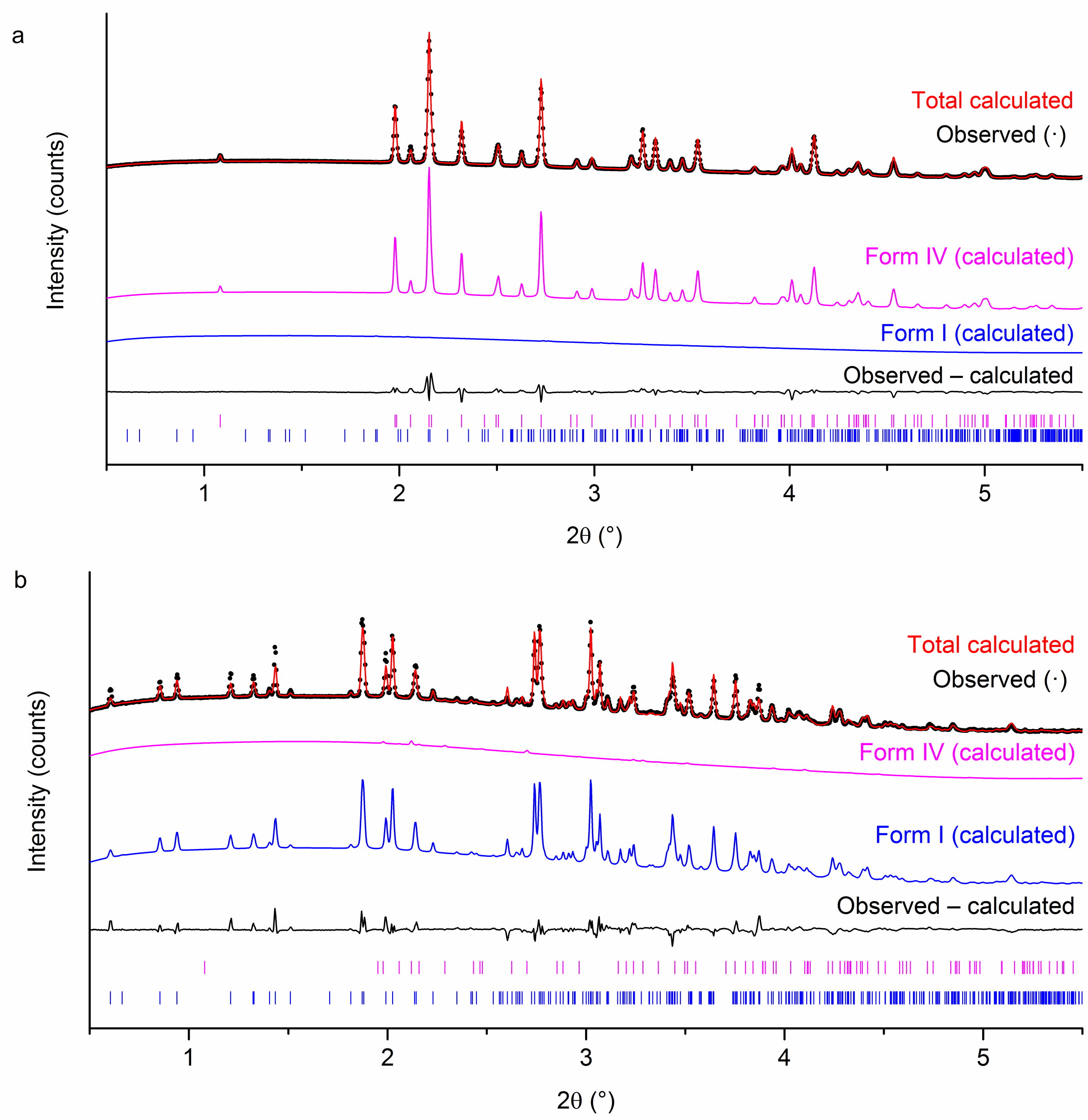

Figure S8. Rietveld refinement data for diffraction patterns recorded at (a) 52 °C and (b) 189 °C when heating a crystalline sample of carbamazepine IV from 52 °C to 217 °C in a DSC. Tick marks show the positions of allowed reflections of carbamazepine form IV (upper) and form I (lower).

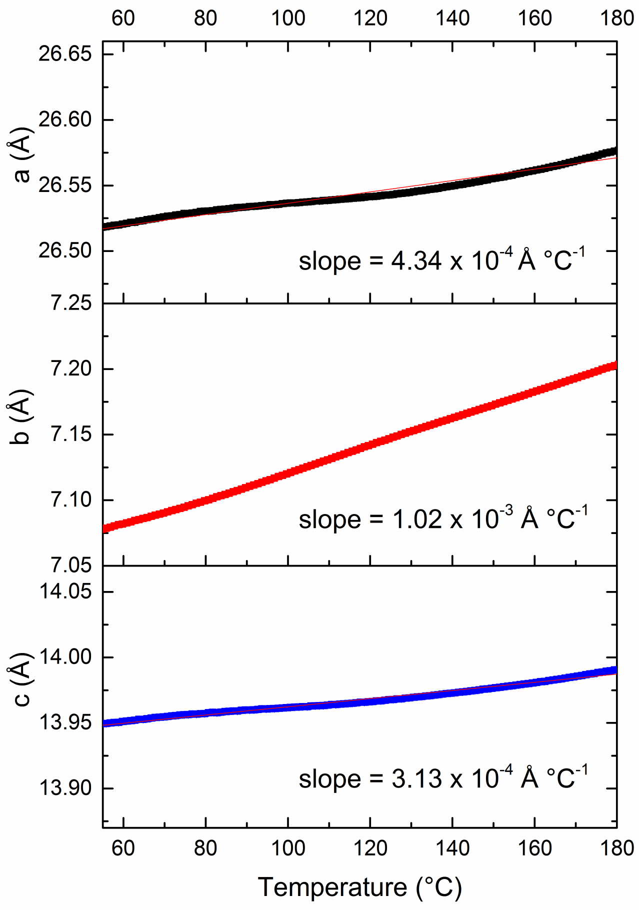

Figure S9. Lattice constants as a function of temperature for carbamazepine IV. Each of the three x-axes are depicted on the same scale to allow for easy comparison.

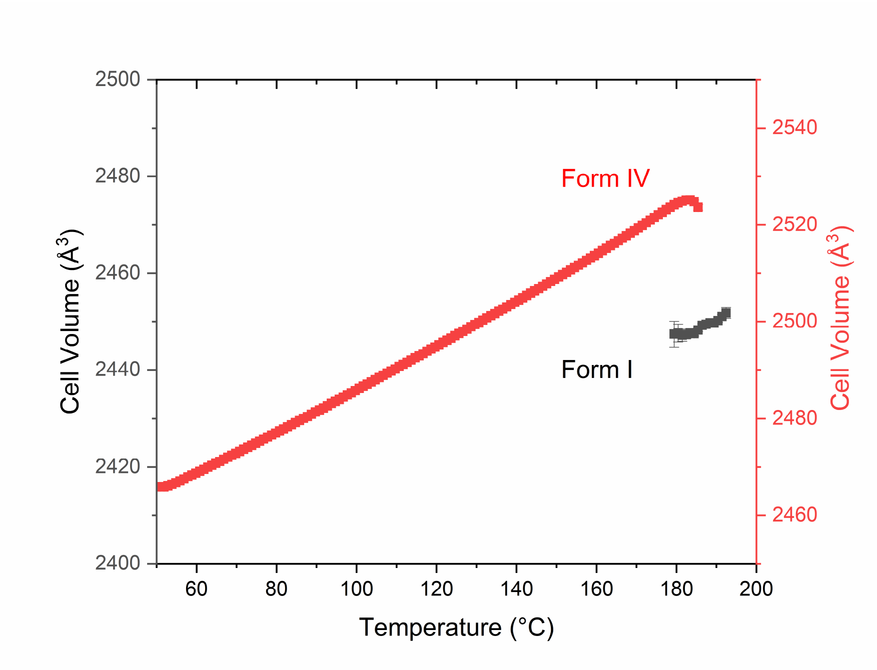

Figure S10. Cell volume as a function of temperature for carbamazepine polymorphs I (black) and IV (red).

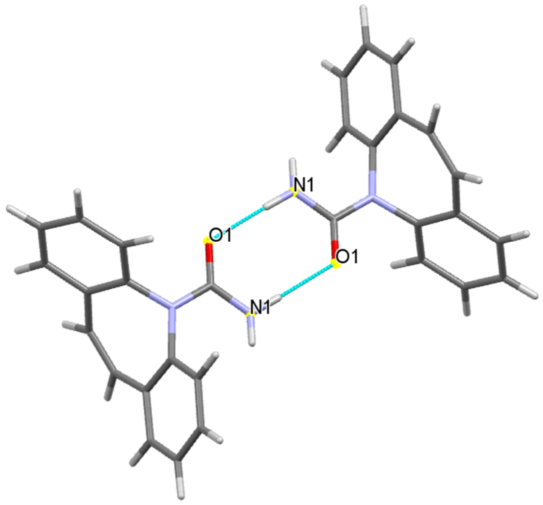

Figure S11. Graphical representation of a CBZ IV dimer, showing hydrogen bonding.

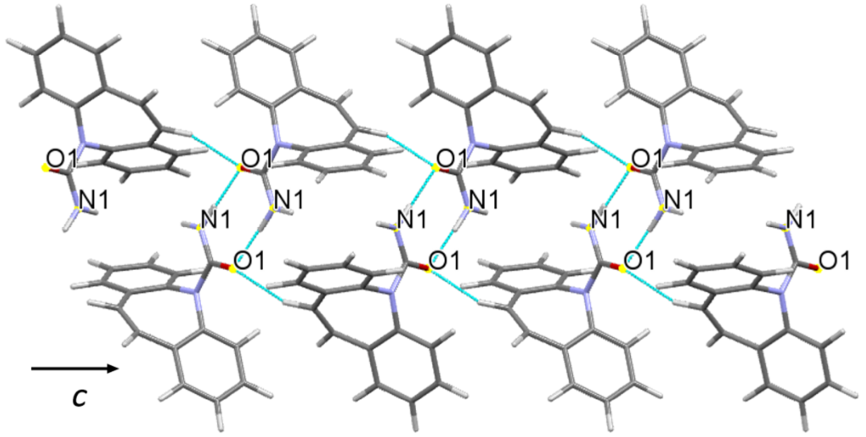

Figure S12. Intermolecular bonding in CBZ IV along the *c* axis of the unit cell, showing N—H···O H-bonds within dimers and C—H···O interactions between dimers.

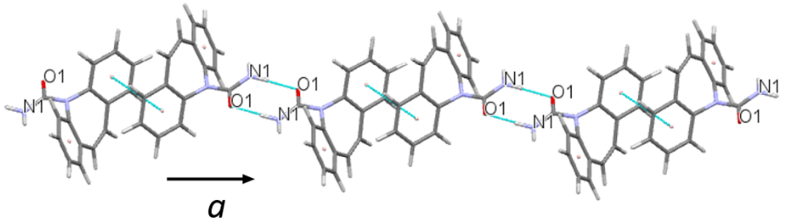

Figure S13. Intermolecular bonding in CBZ IV along the *a* axis of the unit cell, showing N—H···O H-bonds within dimers and centroid···centroid interactions between dimers.

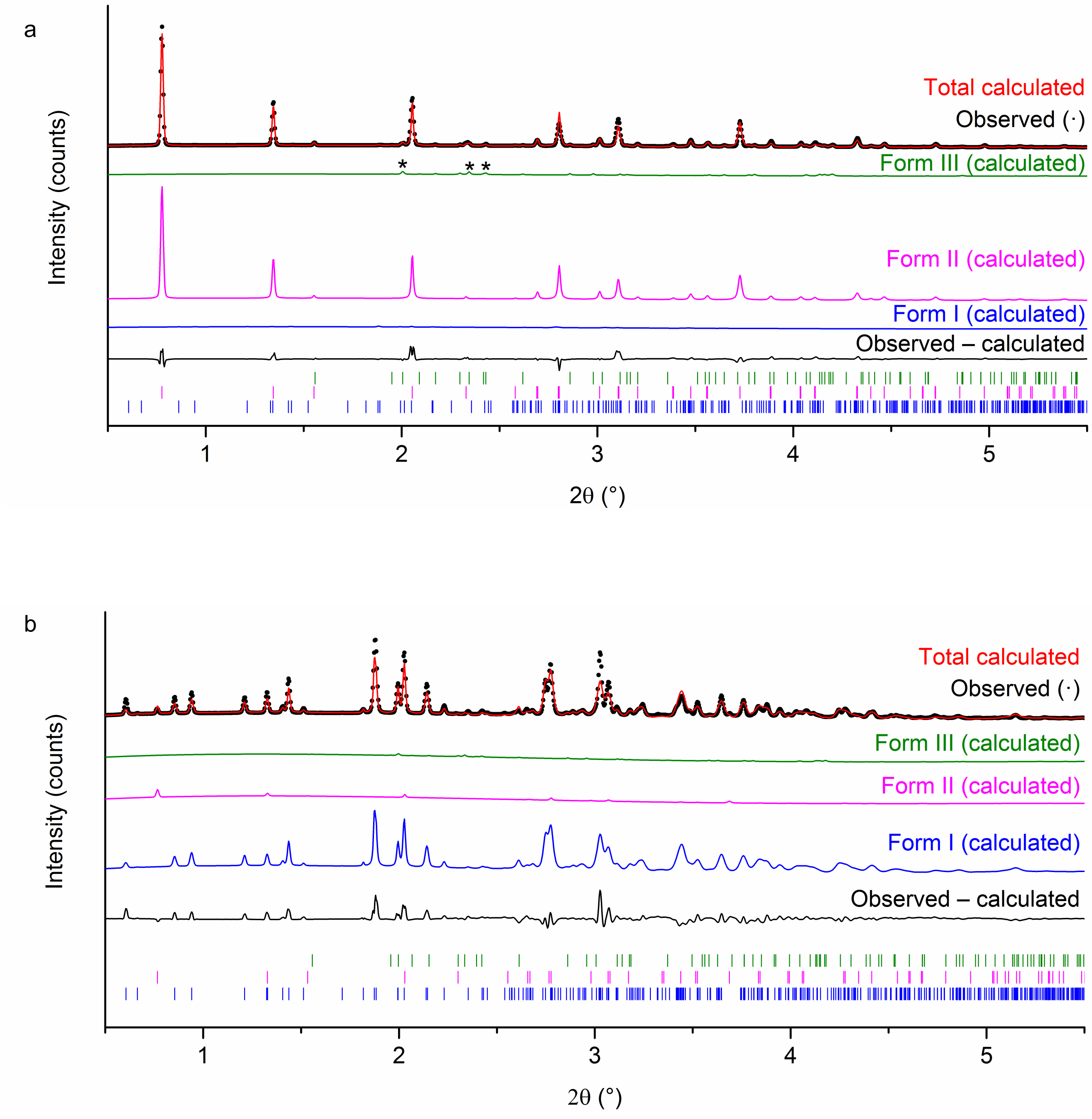

Figure S14. Powder X-ray diffraction patterns for carbamazepine II at (a) 42 °C and (b) 178 °C, including calculated patterns for the contribution of each polymorph. The tick marks show the positions of allowed reflections from carbamazepine form I (blue), form II (pink) and form III (green) and the asterisks denote characteristic reflections of form III.

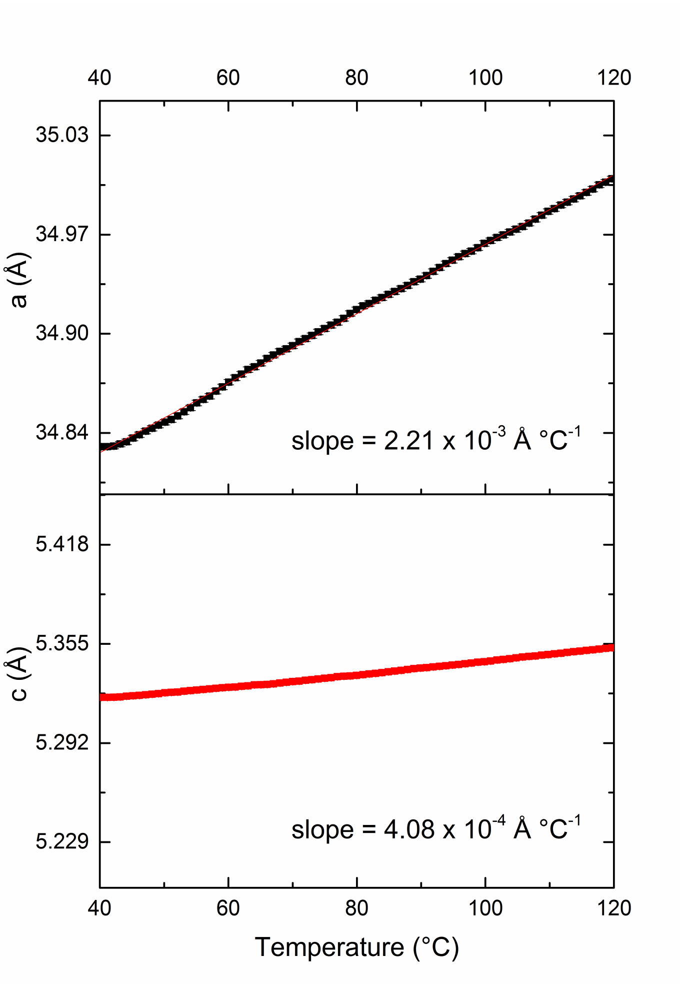

Figure S15. Lattice constants as a function of temperature for carbamazepine form II.

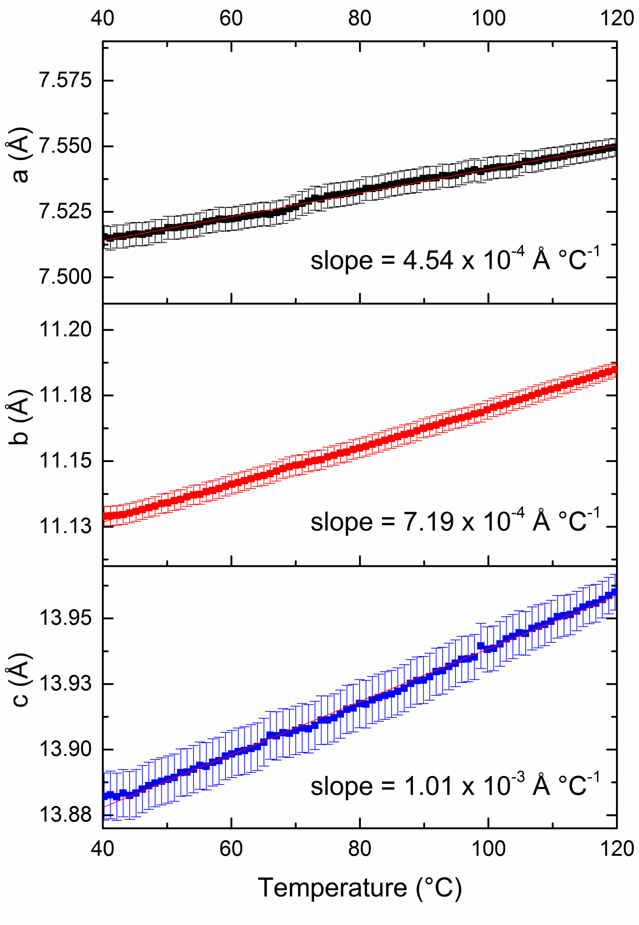

Figure S16. Lattice constants as a function of temperature for carbamazepine form III.

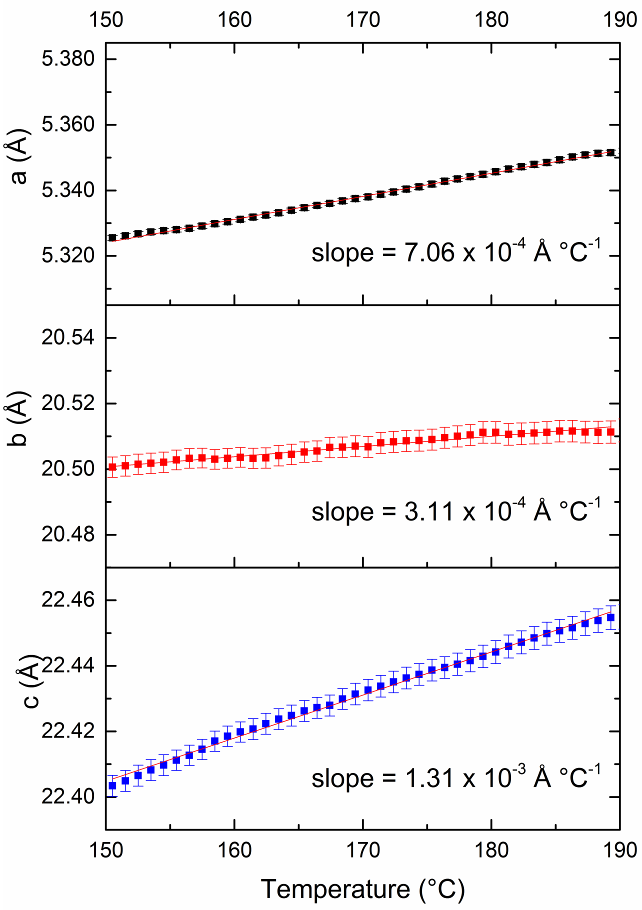

Figure S17. Lattice constants as a function of temperature for carbamazepine form I.

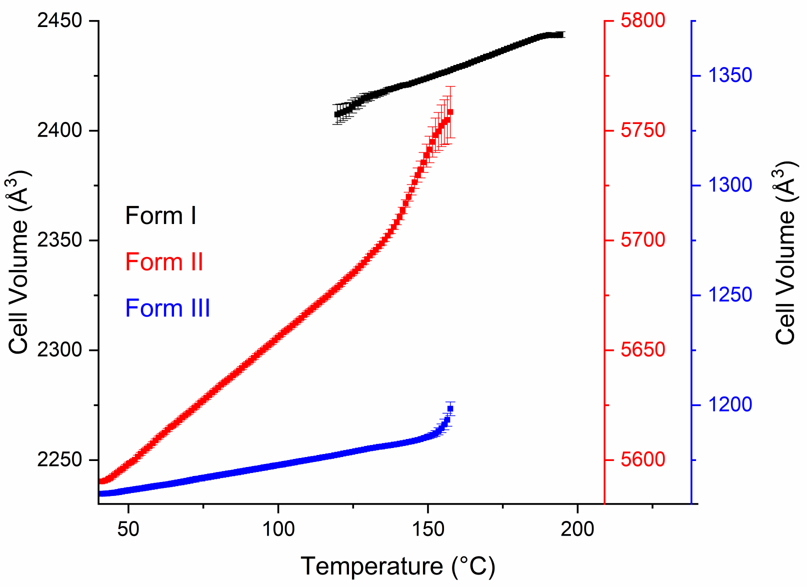

Figure S18. Cell volume as a function of temperature for carbamazepine polymorphs I (black), II (red), and III (blue).
